# Zinc Homeostasis in Bone: Zinc Transporters and Bone Diseases

**DOI:** 10.3390/ijms21041236

**Published:** 2020-02-12

**Authors:** Tongling Huang, Guoyong Yan, Min Guan

**Affiliations:** 1 Center for Human Tissues and Organs Degeneration, Institute of Biomedicine and Biotechnology, Shenzhen Institutes of Advanced Technology, Chinese Academy of Sciences, Shenzhen 518055, Guangdong, China; tl.huang@siat.ac.cn (T.H.); gy.yan@siat.ac.cn (G.Y.); 2 Shenzhen College of Advanced Technology, University of Chinese Academy of Sciences, Shenzhen 518055, Guangdong, China

**Keywords:** zinc, ZIP, ZnT, bone homeostasis, bone diseases, osteogenesis, osteoclastogenesis

## Abstract

Zinc is an essential micronutrient that plays critical roles in numerous physiological processes, including bone homeostasis. The majority of zinc in the human body is stored in bone. Zinc is not only a component of bone but also an essential cofactor of many proteins involved in microstructural stability and bone remodeling. There are two types of membrane zinc transporter proteins identified in mammals: the Zrt- and Irt-like protein (ZIP) family and the zinc transporter (ZnT) family. They regulate the influx and efflux of zinc, accounting for the transport of zinc through cellular and intracellular membranes to maintain zinc homeostasis in the cytoplasm and in intracellular compartments, respectively. Abnormal function of certain zinc transporters is associated with an imbalance of bone homeostasis, which may contribute to human bone diseases. Here, we summarize the regulatory roles of zinc transporters in different cell types and the mechanisms underlying related pathological changes involved in bone diseases. We also present perspectives for further studies on bone homeostasis-regulating zinc transporters.

## 1. Introduction

Bone is the main supporting framework of the body and is characterized by its nature of rigidity and self-healing ability [1]. It provides protection for soft organs and a stable environment for the marrow, which is responsible for hemopoiesis and fat storage [2,3]. Bone is a highly dynamic tissue that undergoes constant physiological activities to remove old, injured or microdamaged bone and replace it with newly formed bone that is mechanically stronger in promoting or preserving bone strength and adapting to changing biomechanical forces [4]. Healthy bones are always in homeostasis, and disturbance of homeostasis causes various bone diseases, such as osteoporosis and osteoarthritis (OA) [5,6,7]. Bone homeostasis involves multiple coordinated cellular and molecular events that revolve around three main types of bone cells: osteoblasts, osteocytes and osteoclasts [8]. Osteoblasts are large cuboidal cells that lie along the bone surface and are responsible for osteogenesis, contributing to bone matrix synthesis and subsequent mineralization [9]. Osteoblasts produce and secrete cellular products during bone formation, are trapped in lacunae and eventually transform into osteocytes [10]. Osteocytes are the most abundant cell type in bone, and they serve as the primary mechanosensor and respond to hormone cues [11,12]. Osteoclasts are large multinucleated cells that are responsible for osteoclastogenesis, accounting for bone destruction and resorption [13]. Osteoblasts originate from mesenchymal stem/stromal cells, whereas osteoclasts are derived from monocyte fusion and have more than one nucleus [14]. Chondrocytes are another type of cell that is very important for bone; they are the only cell type found in the cartilage and are responsible for cartilaginous matrix production and maintenance [15]. Bone homeostasis is synergistically regulated by a series of regulatory factors and sophisticated signaling pathways [16,17,18].

Bone itself is also an important reservoir for diverse metal ions, cytokines, and growth factors [19]. As an essential metal element, zinc plays indispensable roles in the growth and development of humans and other animals [7,20,21,22]. In humans, bone contains approximately 30% of the total body zinc, which has been shown to decrease during aging [23,24]. Zinc deficiency caused by different pathological conditions was found to give rise to bone growth retardation, suggesting that zinc is of great importance for the development, growth, and health of bone [25]. The majority of zinc in the body is stored in the skeleton and enriched in the osteoid layer, where inorganic mineral salts are deposited [26]. Specifically, zinc is found to occur in the mineral component, probably in hydroxyapatite or in complex with fluoride, which might improve the crystallinity of apatite [27,28,29]. In addition to being one of the components of bone, zinc also acts as an activator or coactivator of a variety of proteins involved in bone homeostasis in the form of zinc fingers or zinc clusters [30,31,32]. For instance, zinc acts as a coactivator of runt-related transcription factor 2 (Runx2), which is the earliest determinant of osteogenic differentiation, and its downstream target gene Osterix, which is itself a zinc finger motif-containing transcription factor [25,33].

There has been growing interest in the potential roles of zinc in bone homeostasis and bone diseases. The transport of zinc through cellular and intracellular membranes is of great significance for maintaining zinc homeostasis, suggesting that homeostasis-regulating zinc transporters could be very important in the regulation of physiological and pathological processes in bone. In mammals, there are two types of zinc transporter genes: the *Slc39a* family of importers encodes Zrt- and Irt-like proteins (ZIPs), and the *Slc30a* family of exporters encodes zinc transporters (ZnTs). The ZIP family mediates zinc influx from extracellular fluid or intracellular vesicles into the cytoplasm, and the ZnT family promotes zinc efflux from cells or influx into intracellular vesicles from the cytoplasm. In mammalian genomes, a total of 14 members of the ZIP family (ZIP1–ZIP14) and 10 members of the ZnT family (ZnT1–ZnT10) have been identified [34]. Most ZIP proteins are homo- or heterodimers with eight transmembrane domains, and the N-terminal and C-terminal regions are located extracellularly, with a histidine-rich loop between the third and fourth transmembrane domains [23,35,36,37]. ZnT transporters have six transmembrane domains with their termini inside the cytoplasm, and there is a long histidine-rich loop between transmembrane domains IV and V, which is the binding site of zinc ions [34,38]. Herein, we review the biological functions of six members of the ZIP family and two members of the ZnT family that play regulatory roles in bone homeostasis, the imbalance of which might lead to certain bone diseases (Table 1).

## 2. Roles of ZIP Transporters in Bone

### 2.1. ZIP1, ZIP2 and ZIP3

Zrt/Irt-like proteins 1, 2 and 3 (ZIP1, ZIP2, and ZIP3) are multipass membrane zinc transporter proteins that are responsible for mammalian zinc homeostasis by mediating zinc uptake into the cytoplasm [58,59]. Osteoblasts are known to be the primary functional cells for bone formation. ZIP1 was found to be expressed early in mouse embryo development, at E15.5 in osteoblasts and later in ameloblasts and odontoblasts, indicating that ZIP1 is closely related to bone formation [60]. Additionally, ZIP1 is located in the cytoplasmic membrane and mediates zinc influx during mesenchymal stem cell (MSC) differentiation into osteoblasts [40]. ZIP1 expression was induced upon osteogenic differentiation of multipotent human MSCs and osteoblast progenitor MC3T3-E1 cells in vitro, accompanied by an increase in intracellular zinc uptake [39,40,41]. Moreover, ZIP1-mediated zinc influx gave rise to higher expression of the osteogenic master regulators *Runx2* and *Osterix*. Enhancement of Runx2 and Osterix expression further modulated transcriptional expression of *ZIP1* by directly binding to the response elements in the promoter. These processes resulted in a series of intertwined feed-forward loops that induced zinc influx and osteogenic differentiation and promoted bone apatite formation [41,61,62]. On the other hand, ZIP1 is also expressed in osteoclasts, and overexpression of ZIP1 inhibited mature osteoclast activity by exerting a negative impact on the nuclear factor kappa B (NF-κB) pathway, which is essential for osteoclast differentiation and activity [63]. Thus, ZIP1 could play regulatory roles in stimulating osteogenesis and suppressing osteoclastogenesis in vitro. Although ZIP1 is widely expressed in mouse tissues, no visible bone phenotype was observed in *ZIP1* knockout mice fed a normal diet; however, a decreased embryonic survival rate was found upon feeding with a low-zinc diet, but no obvious bone phenotype was revealed [42,43,44].

Unlike that of ZIP1, the expression of ZIP2 and ZIP3 is low in tissues, and few studies have been published on the roles of ZIP2 and ZIP3 in osteogenesis or osteoclastogenesis in vitro. Although ZIP2 expression has been reported to correlate with hormones, no obvious bone phenotypes appeared in homozygous *ZIP2* knockout mice with an adequate dietary zinc supply [43,44,45]. Similarly, *ZIP3* knockout mice; double *ZIP1* and *ZIP3* knockout mice; and even triple *ZIP1*, *ZIP2*, and *ZIP3* knockout mice exhibited no differences in the content of bone zinc and no bone deformity, but the triple knockout mice exhibited a higher risk of malformations under the condition of dietary zinc deficiency [42,43,44]. These interesting phenomena imply that there might be other compensatory pathways that contribute to the process of zinc uptake in vivo.

### 2.2. ZIP8

Zrt/Irt-like protein 8 (ZIP8) is an 8-transmembrane transporter that is present on the cytoplasmic membrane or intracellular vesicles of various types of cells [64,65]. Zinc supplementation or overexpression of ZIP8 in osteoblasts could improve zinc homeostasis, which helps to protect against intermittent hypoxia (IH) exposure in vitro [66]. IH-treated osteoblasts exhibited reductions in mineralization and zinc content, accompanied by decreased expression of *ZIP8*, *Runx2*, *Col1α1*, and other osteogenesis-associated genes, which could be recovered by zinc supplementation or ZIP8 overexpression [66]. Notably, IH-treated animals displayed significantly impaired insulin production and bone fragility due to an imbalance of zinc homeostasis [67,68].

Recently, ZIP8-mediated zinc influx has been revealed to be essential for the pathogenesis of osteoarthritis [46]. OA is characterized by cartilage degradation, synovitis, osteophyte formation, and sclerosis of subchondral bone. Tissue destruction is caused by proteins named matrix-degrading enzymes, which are produced by cartilage cells. Matrix-degrading enzymes need zinc to function, implying that zinc levels play an important role in OA [69]. ZIP8 expression was found to be increased in chondrocytes from OA patients or OA mouse models, resulting in increased intracellular zinc concentrations [46,47,70]. ZIP8 is embedded in the plasma membrane of cartilage cells and is involved in transporting zinc into these cells from the outside environment. Mechanistically, zinc influx through ZIP8 activates the nuclear localization of a transcription-activating protein, metal-regulatory transcription factor-1 (MTF1), which in turn upregulates the expression of zinc-dependent metalloprotease matrix-degrading enzymes (MMP3, MMP9, MMP12, MMP13) and ADAM metallopeptidase with thrombospondin type 1 motif 5 (ADAMTS5), induces the destruction of the cartilage extracellular matrix and exacerbates the pathogenesis of OA [46,48,71]. Moreover, overexpression or transgenic manipulation of *ZIP8* or *MTF1* in chondrocytes causes OA pathogenesis in mice. It is worth noting that the pathogenesis of OA caused by overexpression of ZIP8 was significantly inhibited in *MTF1*-deficient mice, whereas the pathogenesis of OA caused by overexpression of MTF1 was not affected in *ZIP8*-deficient mice. This clearly shows that MTF1 is a downstream mediator of ZIP8 in the pathogenesis of OA. In contrast, chondrocyte-specific deficiency of *ZIP8* or *MTF1* ameliorated cartilage destruction in OA mice [46]. Furthermore, the zinc-ZIP8-MTF1 axis could reciprocally activate hypoxia-inducible factor 2α (HIF-2α), one of the key regulatory factors of endochondral osteogenesis and OA-related cartilage degeneration, to amplifying catabolic signals and accelerating cartilage destruction [49,72,73]. Therefore, the zinc-ZIP8-MTF1 axis might be a novel drug target for the treatment of OA.

### 2.3. ZIP13

Zrt- and Irt-like protein 13 (ZIP13) possesses an 8-transmembrane structure and acts as a zinc-influx transporter, moving zinc from the subcellular compartment into the cytosol [74]. ZIP13 is located in the Golgi apparatus and intracellular vesicles and mainly plays roles in mesenchyme-originating cells [51,74,75,76]. ZIP13 can respond to and be regulated by zinc concentrations. This process might contribute to the influx of stored zinc from vesicles to the cytosol, meeting the requirement for zinc in order to activate enzymes and related metabolic reactions under zinc-restricted conditions [76,77]. ZIP13 was found to be expressed in osteoblasts of the tibia and alveolar bone, in the proliferative zone of the growth plate, in odontoblasts of the forming dentin crown in molars, and in fibroblasts of the reticular layer of the skin [51,78,79].

The spondylocheiro dysplastic form of Ehlers–Danlos syndrome (SCD-EDS) is an inherited connective tissue and bone disease caused by a homozygous mutation of the *ZIP13* gene [50,77]. Zinc deficiency can cause bone growth retardation and increase the risk of skin fragility [80,81]. Many clinical features of SCD-EDS patients are similar to symptoms of zinc deficiency in humans or animal models, such as osteogenesis imperfecta, skin inelasticity, and systemic growth retardation, which is also recapitulated in *ZIP13*-knockout mice [50,51]. Mice with *ZIP13* deletion exhibited impaired bone morphogenetic protein 4 (BMP4)/transforming growth factor β (TGF-β)/Smad signaling in the corresponding tissues. Bone morphogenetic proteins and TGF-β signaling are known to play important roles in cell proliferation and differentiation and are highly correlated with the development of bone and various tissues [82]. Ablation of ZIP13 initiated dysregulation of a variety of differentiation-related marker genes leading to inadequate maturation of osteoblasts, chondrocytes, odontoblasts and fibroblasts; these markers included 1) *Runx2* and Msh homeobox 2 (*Msx2*), which are involved in skeletogenesis; 2) Indian hedgehog (*Ihh*), Fibroblast growth factor receptor 3 (*Fgfr3*) and SRY-box transcription factor 9 (*Sox9*), which are responsible for differentiation of chondrocytes; and 3) Collagen type I alpha 2 (*Col1α2*), which is responsible for fibroblast differentiation [51,75]. Furthermore, the valosin-containing protein (VCP)-dependent ubiquitin proteasome pathway accelerates the degradation of the pathological mutant ZIP13 protein, reduces the level of functional protein, and leads to vertebral dysplasia in SCD-EDS [78,83].

### 2.4. ZIP14

ZIP14 is most closely related to ZIP8, which is known to be localized to the plasma membrane and to function in the transport of Zn^2+^, Fe^2+^, and Mn^2+^ [84,85,86]. ZIP14 is important for mammalian development and was found to be highly expressed in the pituitary gland and bone, which are responsible for the production of growth hormone and bone elongation [52,53]. ZIP14 is highly expressed in chondrocytes in the proliferation zone of growth plates and contributes to endochondral ossification, which accounts for longitudinal lengthening of bones. Loss of *ZIP14* in mice results in dwarfism, scoliosis, osteopenia and shortening of the long bones caused by abnormal chondrogenesis and endochondral ossification [52,53]. At the same time, intracellular zinc concentrations in the growth plate proliferation zone as well as zinc concentrations and 3′,5′-Cyclic adenosine monophosphate (cAMP) levels in pituitary cells of ZIP14 knockout mice were significantly reduced, and these effects were important for maintaining the homeostasis of G protein-coupled receptor (GPCR)-mediated signal transduction. Further research on the underlying mechanisms revealed that zinc transport mediated by ZIP14 could prevent cAMP degradation by inhibiting phosphodiesterase (PDE) activity, through which ZIP14 facilitates the GPCR/cAMP/cAMP response element-binding protein (CREB) signaling pathways [52,53]. The expression of ZIP14 is stable during osteogenic differentiation of mouse MSCs, whereas it increases during the mineralization stage. *ZIP14* knockout mice exhibited markedly reduced trabecular bone mass that was greatly amplified with aging [87]. In humans, hyperostosis cranialis interna (HCI) is a genetic disease related to a missense mutation (P.L441R) in the *ZIP14* gene, which was confirmed by whole-exome sequencing [88]. P.L441R-ZIP14 leads to incorrect localization of ZIP14 in osteoblasts, thus increasing the accumulation of unstable cellular zinc and abnormal cellular zinc homeostasis, causing excessive bone growth in the skull [54]. Notably, ZIP14L438R osteoblast-specific knock-in mice, which have a missense mutation in the mouse *ZIP14* gene, exhibit no effects on the calvariae but have a severe femoral phenotype characterized by a sharp increase in cortical thickness, similar to the underlying pathology of HCI patients. Conditional osteoblast *ZIP14*-mutation knock-in mice have a significant increase in cortical thickness and a decreased midshaft due to enhanced intimal formation caused by significantly enhanced cAMP/CREB and nuclear factor of activated T cells (NFAT) signaling activity, which plays an important role in bone and inflammatory processes; however, conditional osteoclast ZIP14-mutation knock-in mice display no significant difference in skeletal phenotype.

## 3. Role of ZnT Transporters in Bone

The zinc transporter (ZnT)/Slc30 family is a subfamily responsible for zinc efflux, in contrast to the function of the (ZIP)/Slc39 subfamily [89]. Few studies have been reported on the relationship between the ZnT family and bone, except for ZnT5 and ZnT7. ZnT5 expressed after transfection in HeLa cells was reported to localize to Golgi membranes, mediating zinc efflux from the cytosol to the Golgi compartment [90]. ZnT7 is localized to the Golgi apparatus and unique vesicular compartments [91]. The activity of tissue-nonspecific alkaline phosphatase (TNAP) was significantly reduced in DT40 cells in the absence of both ZnT5 and ZnT7 [92,93]. As a zinc-requiring membrane-bound enzyme, TNAP is crucial for bone mineralization. Decreased TNAP activity inevitably leads to impaired mineralization of the bone matrix, which mimics the skeletal defects of infantile hypophosphatasia [94]. Mice with *ZnT5* deficiency displayed poor growth, osteopenia and heart failure [55]. Specifically, *ZnT5* knockout mice manifest bone abnormalities, including lower bone density due to impairment of osteoblast maturation [55]. However, the roles of ZnT5 in bone and the related mechanisms are largely elusive. The ZnT7 protein is abundantly expressed in mouse osteoblast MC3T3-E1 cells [57] and is a crucial component of the functional activities of MSCs [56]. ZnT7 prevented MC3T3-E1 cells from undergoing apoptosis induced by oxidative stress by reducing the accumulation of intracellular cationic zinc [57]. On the other hand, the expression of ZnT7 decreased upon osteogenic induction of MSCs. Downregulation of ZnT7 expression by small interfering RNA (siRNA) increased the levels of osteogenesis-associated markers such as alkaline phosphatase (ALP), collagen I and osteocalcin, whereas overexpression of ZnT7 exerted the opposite effects. Further study demonstrated that ZnT7 could interfere with Wnt/β-catenin signaling, a key pathway involved in osteogenesis [56]. ZnT5 and ZnT7 might play opposite roles in osteogenesis; however, the underlying mechanism still needs to be explored.

## 4. Conclusions and Future Prospects

**Figure 1:**
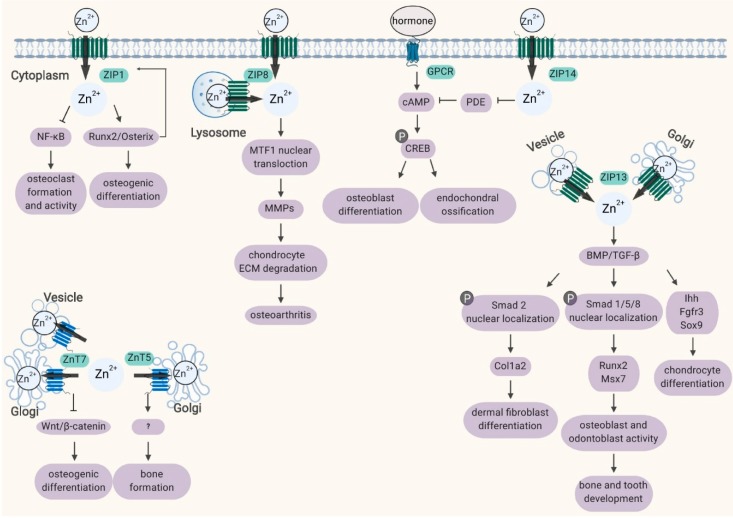
Schematic diagram of the subcellular localization and biological processes of ZIP and ZnT proteins involved in bone homeostasis. Refer to the text for a detailed description. Abbreviations: MTF1, metal-regulatory transcription factor-1; MMPs, matrix metalloproteinases; GPCR, G protein-coupled receptor; PDE, phosphodiesterase; CREB, cAMP response element-binding protein; BMP, bone morphogenetic protein.

Zinc is an important element for bone development, regeneration, and homeostasis, which has been gradually recognized in recent years [95]. However, zinc is incapable of passing through the cell membrane freely, and transporter-mediated influx and efflux of zinc are required for zinc homeostasis in cells. Since the discovery of zinc transporters, accumulating evidence has demonstrated that mutation and disorder of ZIP and ZnT transporter proteins are related to certain aspects of bone physiology or disease [96]. In this review, eight zinc transporters, namely, ZIP1, ZIP2, ZIP3, ZIP8, ZIP13, ZIP14, ZnT5, and ZnT7, were characterized to illuminate the relationship between zinc transporters and bone homeostasis (Figure 1). Different members of the ZIP and ZnT families synergize with zinc to participate in the processes of osteogenesis and osteoclastogenesis. The ZIP8-mediated zinc signaling axis in chondrocytes is an essential regulator of OA pathogenesis; dysfunction of the *ZIP13*, *ZIP14*, or *ZnT5* gene gives rise to bone deformity in mice and humans. Mutations in zinc transporter genes may lead to genetic disease or increase the risk of disease. However, studies related to the role of zinc transporters in the pathogenesis of bone diseases are relatively rare, and further investigation is needed to gain a deeper understanding, which might facilitate the development of zinc supplements for bone diseases, zinc-containing bone repair materials, and molecular therapies targeting zinc signaling pathways. In addition, recent studies suggest that zinc transporters can transport not only zinc but also trace elements such as iron, manganese, and cadmium, which makes the functions of zinc transporters in bone more complex and diverse. Whether a specific zinc transporter transports other divalent metal ions, whether the same zinc transporter regulates bone development by coordinating the steady-state metabolism of different trace elements, and whether the transport function of a zinc transporter for multiple trace elements is independent or balanced are important scientific issues to be further studied.

## Figures and Tables

**Table 1:** Zrt- and Irt-like protein (ZIP) and zinc transporter (ZnT) proteins in bone physiology and pathology.

Name	Mutation Type	Physiological Events and Diseases
ZIP1		induction of osteogenesis of MSCs [39,40,41]
	knockout	no obvious bone phenotype [42,43,44]
ZIP2	knockout	no obvious bone phenotype [43,44,45]
ZIP3	knockout	no obvious bone phenotype [42,43,44]
ZIP8		cartilage destruction and osteoarthritis [46,47,48,49]
ZIP13	mutation	spondylocheiro dysplastic form of Ehlers-Danlos syndrome [50]
	knockout	connective tissue dysplasia [51]
ZIP14	knockout	growth retardation [52,53]
	mutation	hyperostosis cranialis interna [54]
ZnT5	knockout	poor growth, osteopenia and heart failure [55]
ZnT7		impaired osteogenesis of MSCs [56]
		protection of osteoblasts from apoptosis [57]

## Abbreviations

ZIP	Zrt- and Irt-like protein
ZnT	zinc transporter
MSCs	mesenchymal stem cells
OA	osteoarthritis
MTF1	metal-regulatory transcription factor-1
Runx2	runt-related transcription factor 2
NF-κB	nuclear factor kappa B
ADAMTS5	ADAM metallopeptidase with thrombospondin type 1 motif 5
Msx2	msh homeobox 2
Ihh	Indian hedgehog
Fgfr3	fibroblast growth factor receptor 3
Sox9	SRY-box transcription factor 9
Col1α2	collagen type I alpha 2
MMPs	matrix metalloproteinases
VCP	valosin-containing protein
cAMP	3′,5′-Cyclic adenosine monophosphate
NFAT	nuclear factor of activated T cells
ALP	alkaline phosphatase
siRNA	small interfering RNA
PDE	phosphodiesterase
GPCR	G protein-coupled receptor
CREB	cAMP response element-binding protein
BMP	bone morphogenetic protein
SCD-EDS	spondylocheiro dysplastic form of Ehlers-Danlos syndrome
HCI	hyperostosis cranialis interna
BMP	bone morphogenetic protein
